# Nuclear entry and export of FIH are mediated by HIF1α and exportin1, respectively

**DOI:** 10.1242/jcs.219782

**Published:** 2018-11-19

**Authors:** Yihua Wang, Shan Zhong, Christopher J. Schofield, Peter J. Ratcliffe, Xin Lu

**Affiliations:** 1 Ludwig Institute for Cancer Research Ltd., Nuffield Department of Clinical Medicine, University of Oxford, Old Road Campus, Roosevelt Drive, Oxford OX3 7DQ, United Kingdom; 2 Biological Sciences, Faculty of Environmental and Life Sciences, University of Southampton, Southampton SO17 1BJ, United Kingdom; 3 Department of Chemistry, Chemistry Research Laboratory, University of Oxford, Mansfield Road, Oxford OX1 3TA, United Kingdom; 4 Target Discovery Institute, NDM Research Building, University of Oxford, Old Road Campus, Roosevelt Drive, Oxford OX3 7FZ, United Kingdom

**Keywords:** 2-Oxoglutarate, 2-OG, Dioxygenase inhibitors, FIH, Factor inhibiting HIF, HIF asparaginyl hydroxylase, Hypoxia, Nuclear translocation

## Abstract

Hypoxia plays a crucial role at cellular and physiological levels in all animals. The responses to chronic hypoxia are, at least substantially, orchestrated by activation of the hypoxia inducible transcription factors (HIFs), whose stability and subsequent transcriptional activation are regulated by HIF hydroxylases. Factor inhibiting HIF (FIH), initially isolated as a HIFα interacting protein following a yeast two-hybrid screen, is an asparaginyl hydroxylase that negatively regulates transcriptional activation by HIF. This study aimed to define the mechanisms that govern transitions of FIH between the nucleus and cytoplasm. We report that FIH accumulates in the nucleus within a short time window during hypoxia treatment. We provide evidence, based on the application of genetic interventions and small molecule inhibition of the HIF hydroxylases, that the nuclear localization of FIH is governed by two opposing processes: nuclear entry by ‘coupling’ with HIF1α for importin β1-mediated nuclear import and active export via a Leptomycin B-sensitive exportin1-dependent pathway.

This article has an associated First Person interview with the first author of the paper.

## Abstract (precis)

**Summary:** Nuclear localization of FIH is governed by two opposing processes: nuclear entry by ‘coupling’ with HIF1α for importin β1-mediated nuclear import, and active export via a leptomycin B-sensitive exportin 1 (CRM1)-dependent pathway.

## INTRODUCTION

As solid tumours grow and oxygen becomes limiting, hypoxia triggers cellular and physiological events (Ratcliffe, 2013). Hypoxia-inducible factors (HIFs) are upregulated in response to hypoxic conditions and are key factors in coordinating cellular responses to hypoxia. HIF is an α,β-heterodimer that binds DNA at hypoxia response elements (HREs) containing a core RCGTG sequence (Kaelin and Ratcliffe, 2008). There are three HIFα proteins in higher metazoans, with HIF1α and HIF2α being the most extensively studied. HIF1α and HIF2α are closely related, and both activate HRE-dependent gene transcription. Nevertheless, HIF1α and HIF2α play non-redundant roles with distinct transcriptional targets (Kaelin and Ratcliffe, 2008; Ratcliffe, 2007). Levels of HIFα, but not HIFβ, are strongly regulated by oxygen availability, as is the transcriptional activity of HIF.

As a key regulator of the response of mammalian cells to oxygen deprivation and an important player in the adaptation of tumour cells to a hypoxic microenvironment, regulation of the stability and subsequent trans-activational function of HIFα is of major biomedical importance. Under well-oxygenated conditions, HIFα is hydroxylated at prolyl residues by members of the prolyl hydroxylase domain (PHD) family (Myllyharju, 2013). Hydroxylation of these prolyl residues generates a binding site for the von Hippel-Lindau (pVHL) tumour suppressor protein, which is a component of an ubiquitin E3 ligase complex. As a result, HIFα is polyubiquitylated and subjected to proteasomal degradation when oxygen is available. The PHD proteins belong to the Fe(II)- and 2-oxoglutarate (2-OG)-dependent oxygenase superfamily, whose activity is dependent on oxygen. The kinetic properties of the PHDs enable the rate of HIF hydroxylation in cells to be suppressed by hypoxia. Under low oxygen conditions, or in cells lacking functional pVHL, HIFα accumulates, dimerizes with HIFβ, translocates to the nucleus and transcriptionally activates multiple genes, including genes involved in erythropoiesis, angiogenesis, autophagy and energy metabolism (Kaelin and Ratcliffe, 2008).

Factor inhibiting HIF (FIH), another Fe(II)- and 2-OG-dependent dioxygenase, hydroxylates a conserved asparagine residue within the HIFα C-terminal activation domain (CAD), a post-translational modification that blocks interactions between the HIFα CAD and the transcriptional activator/histone acetyl transferases CBP/p300 (Elkins et al., 2003; Hewitson et al., 2002; Lando et al., 2002a,b; Mahon et al., 2001; McNeill et al., 2002). FIH has multiple other substrates, including members of the ankryin repeat domain (ARD) protein family (Cockman et al., 2006, 2009; Coleman et al., 2007; Janke et al., 2013; Karttunen et al., 2015; Zheng et al., 2008). Because HIFα, ARD-containing proteins and other substrates can be located in different cellular compartments, processes that affect the subcellular location of FIH influence its substrate selection and, subsequently, its biological functions, including the regulation of metabolism (Peng et al., 2012a; Scholz et al., 2016; Sim et al., 2018; Zhang et al., 2010), keratinocyte differentiation (Peng et al., 2012b), vascular endothelial cell survival (Kiriakidis et al., 2015), tumour growth (Kuzmanov et al., 2012; Pelletier et al., 2012) and metastasis (Kang et al., 2017) as well as Wnt signalling (Rodriguez et al., 2016).

FIH is ubiquitously expressed in most types of cultured cells and human tissues (Bracken et al., 2006; Stolze et al., 2004). In live cells, overexpressed eGFP-tagged FIH is primarily observed in the cytoplasm, with a low level in the nucleus (Metzen et al., 2003). Consistent with this observation, immunofluorescence analyses of endogenous FIH in cultured HEK 293T cells detected FIH protein predominantly in the cytoplasm (Linke et al., 2004; Stolze et al., 2004). A wide range of human tissues analysed by immunofluorescence also manifested mostly cytoplasmic staining, but for cell types expressing notably high levels of endogenous FIH, nuclear staining was also observed (Soilleux et al., 2005). In clinicopathological studies of human cancer, nuclear localization of FIH was reported to be a positive factor associated with good prognosis. This observation was independent of other more conventional features, including histopathological grading, tumour size and spread to lymph nodes (Kroeze et al., 2010; Tan et al., 2007). Understanding the factors regulating FIH localization is therefore of both biological and medical interest. A previous study reported that hypoxia induces nuclear FIH (Liang et al., 2015), but the underlying mechanism remains unknown. Here, we report a detailed time course analysis of the effects of hypoxia and HIF hydroxylase inhibition on FIH localization. The results reveal that FIH accumulates in the nucleus after exposure to hypoxia within a short timeframe, and that FIH enters and exits the nucleus via HIF1α/importin β1- and Leptomycin B-sensitive exportin1 (CRM1)-dependent pathways, respectively.

## RESULTS

### Hypoxia induces nuclear entry of FIH

**Fig. 1.**
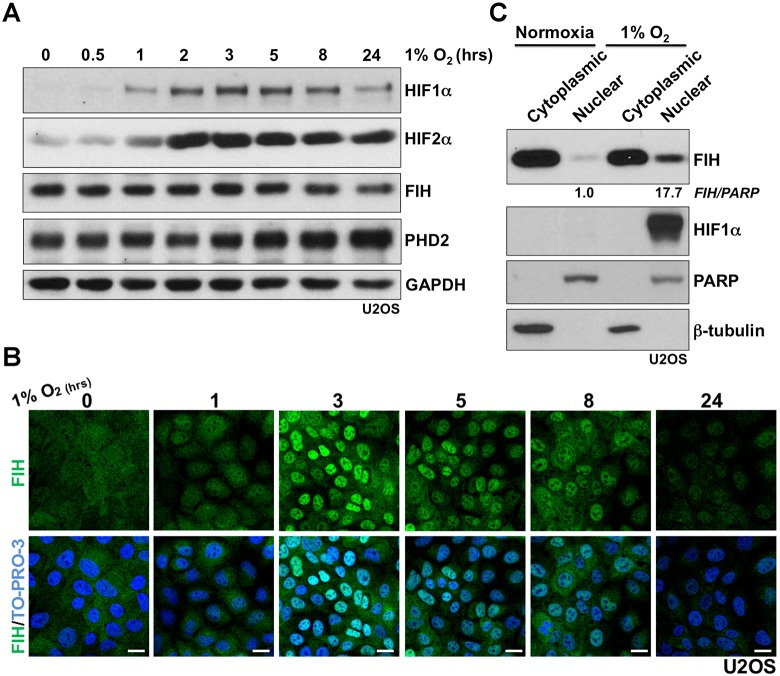
**Evidence that hypoxia induces nuclear entry of FIH.** (A) Protein levels of HIF1α, HIF2α, FIH and PHD2 in U2OS cells during hypoxia (1% O_2_) treatment at the indicated time points. GAPDH was used as a loading control. (B) Immunofluorescence staining of FIH (green) in U2OS cells under hypoxic conditions (1% O_2_) at the indicated time points. TO-PRO-3 (blue) was used to stain nuclei. (C) Protein levels of FIH and HIF1α from cytoplasmic or nuclear fractions in U2OS cells in normoxia or hypoxia (1% O_2_, 3 h). β-tubulin and PARP were used as loading controls for the cytoplasmic and nuclear fractions, respectively. Figures beneath lanes 2 and 4 indicate relative intensities of nuclear FIH in normoxia and hypoxia. Note that different quantities of cytoplasmic and nuclear extracts were loaded. Scale bars: 20 µm.

To investigate whether FIH nuclear import is regulated by hypoxia, a detailed time course of hypoxia (1% atmospheric O_2_) treatment was performed using human osteosarcoma U2OS cells. As shown in Fig. 1A, an overall increase in HIF1α protein level manifests after 1 h of hypoxia treatment, with maximal induction being observed by western blot at about 3 h under hypoxia. After 8 h under hypoxia, an apparent decrease in the level of HIF1α protein was observed. The decrease could be caused by upregulation of prolyl hydroxylase domain-containing protein PHD2 or other PHD isoforms, which are HIF targets (Epstein et al., 2001; Marxsen et al., 2004); PHD2 is a major regulator of HIF1α steady state levels in many cells (Berra et al., 2003; Epstein et al., 2001). Unlike the transient increase in HIF1α, an increase in HIF2α protein level was observed and sustained under hypoxia for at least 24 h. The FIH total protein level was not altered during hypoxia within the limits of detection. However, immunofluorescence studies revealed a striking change in FIH localization in response to hypoxia treatment; clear nuclear accumulation of FIH was observed after 3 h of hypoxia treatment (Fig. 1B). FIH remained localized in the nucleus for several hours, but nuclear FIH was greatly reduced after 24 h (Fig. 1B). FIH immunofluorescence staining was specific because no signal was detected in FIH siRNA-transfected U2OS cells in either normoxia or hypoxia (Fig. S1A). Similar results concerning the effects of hypoxia on FIH localization were obtained with both human colon cancer cells HKe3 (Wang et al., 2014, 2010) and human breast cancer cells MCF-7 (Fig. S1B,C). The hypoxia-induced nuclear accumulation of FIH observed by immunofluorescence staining was further supported by a subcellular fractionation assay that detected a >17-fold increase in FIH protein in the nuclear fractions in U2OS cells after 3 h of hypoxia treatment (Fig. 1C). HIF1α was only present in the nuclear fraction under hypoxia.

### Nuclear entry of FIH is mainly HIF1α-dependent, and requires inhibition of FIH enzyme activity

**Fig. 2.**
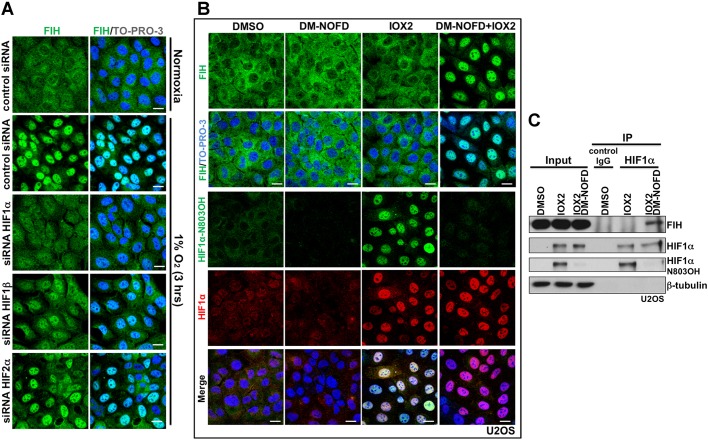
**Nuclear entry of FIH is mainly HIF1α-dependent, and requires inhibition of FIH enzymatic activity.** (A) U2OS cells were transfected with the indicated siRNAs for 3 days, followed by culture in normoxia (20% O_2_) or hypoxia (1% O_2_, 3h). Images show immunofluorescence staining of FIH (green) in U2OS cells after the indicated treatments. TO-PRO-3 (blue) was used to stain nuclei. (B) Immunofluorescence staining of FIH (green), HIF1α N803OH (green) and HIF1α (red) in U2OS cells treated with DMSO, DM-NOFD (1 mM), IOX2 (0.25 mM) or DM-NOFD (1 mM) plus IOX2 (0.25 mM) for 3 h. TO-PRO-3 (blue) was used to stain nuclei. (C) Protein levels of FIH, HIF1α and HIF1α N803OH in U2OS cells after the indicated treatments. β-tubulin was used as a loading control. Total cell lysates from the treated U2OS cells were immunoprecipitated with an anti-HIF1α antibody. Scale bars: 20 µm.

It was reported that, in breast cancer, nuclear FIH expression shows a significant positive correlation with nuclear HIF1α expression (Tan et al., 2007). In the current work, we observed that the time course of induction of HIF1α during hypoxia correlates with nuclear accumulation of FIH. We thus hypothesized that nuclear entry of FIH is dependent on HIF1α. To test this, we depleted HIF1α, HIF2α or HIF1β using siRNAs in U2OS cells, then exposed the cells to hypoxia (Fig. S2A). HIF1α depletion abolished hypoxia-induced nuclear FIH accumulation (Fig. 2A, Fig. S2B). HIF1β depletion also affected hypoxia-induced nuclear FIH, but to a lesser extent than HIF1α (Fig. 2A, Fig. S2B), possibly via downregulation of HIF1α (Fig. S2A) (Chilov et al., 1999). Although the average intensity of nuclear FIH staining decreased following HIF2α depletion, the percentage of nuclear FIH-positive cells did not change significantly (Fig. 2A, Fig. S2B).

To further define the dependence of FIH nuclear localization on the stabilization of HIF1α, we exposed cells to small molecule inhibitors of the HIF hydroxylases with differential selectivity against FIH and the PHDs. To monitor the action of these compounds in cells under the conditions of our experiments, we deployed an antibody that specifically recognizes N803-hydroxylated HIF1α, as assessed by mass spectrometry and reactivity against synthetic peptides (Lee et al., 2008; Tian et al., 2011). As expected, these compounds displayed differential activity against FIH and induced HIF1α in a form that is (IOX2, FG2216, DFO) or is not (DMOG, IOX1, VGB10B/IOX4) hydroxylated on HIF1α N803 (Fig. S2C). Immunostaining of aliquots of the same cells for FIH revealed a striking correlation between the nuclear localization of FIH and conditions in which HIF1α was induced in a form without detectable N803 hydroxylation (Fig. S2D). These results indicated that the hydroxylation status of HIF1α at N803 affects the nuclear localization of FIH. Although HIF1α that was not hydroxylated on N803 could not be measured directly, the findings suggested that the nuclear accumulation of non-hydroxylated HIF1α was responsible for FIH relocation. We postulated that this was a result of increased binding of FIH to unhydroxylated HIF1α, as expected from previous work demonstrating that catalytic inhibitors of FIH promote binding to its substrates (Cockman et al., 2009). To test this more directly, we exposed cells to specific inhibitors of both PHDs (IOX2) (Chan et al., 2016) and FIH (DM-NOFD) (McDonough et al., 2005), or to both compounds in combination (Fig. 2B,C). As expected, IOX2 induced HIF1α in a form that was substantially hydroxylated on N803, whereas additional exposure to DM-NOFD suppressed N803 hydroxylation (Fig. 2B,C). Under these conditions, clear nuclear localization of FIH was observed when IOX2 was combined with DM-NOFD, but not when it was used alone (Fig. 2B). Consistent with the hypothesis that this reflected binding of FIH to unhydroxylated HIF1α, immunoprecipitation revealed that binding of FIH to HIF1α was induced when N803 hydroxylation of HIF1α was suppressed by DM-NOFD (Fig. 2C).

Taken together, these findings suggest that FIH enters the nucleus in association with its substrate, HIF1α, and that this process is enhanced by catalytic inhibition.

### FIH enters and exits the nucleus via HIF1α/importin β1- and Leptomycin B-sensitive exportin1-dependent pathways, respectively

**Fig. 3.**
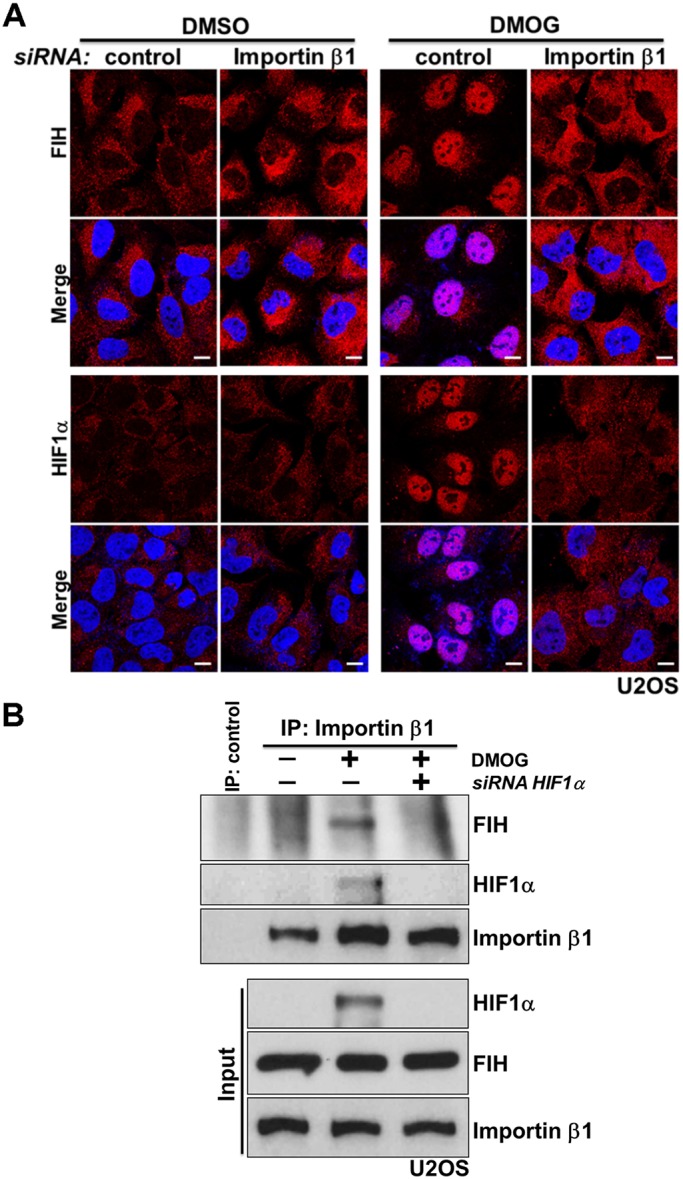
**FIH complexes with importin β1 via HIF1α for nuclear import.** (A) U2OS cells were transfected with control siRNA or importin β1 siRNA for 3 days, followed by treatment with DMSO or DMOG (1 mM) for 3 h. Images show immunofluorescence staining of FIH (red) or HIF1α (red) in U2OS cells after the indicated treatments. DAPI (blue) was used to stain nuclei. (B) U2OS cells were transfected with control siRNA or HIF1α siRNA for 3 days, followed by treatment with DMSO or DMOG (1 mM) for 3 h. Total cell lysates from the U2OS cells were immunoprecipitated with an anti-importin β antibody or control IgG. FIH, HIF1α and importin β levels are indicated. Scale bars: 10 µm.

FIH has 349 residues and forms an ∼80 kDa homodimer in solution (Dann et al., 2002; Elkins et al., 2003), which is essential for its efficient catalysis (Lancaster et al., 2004). The transport of proteins larger than ∼40 kDa between the nucleus and cytoplasm through the nuclear pore complex (NPC) is a spatially and temporally controlled process (Adams and Wente, 2013; Aitchison and Rout, 2012). For nuclear import, target proteins using the classical nuclear import system bind to dimeric complexes of importin α/β proteins (Tran et al., 2014). Observations by Depping et al., (2015) indicate that FIH does not interact with importins α and β. These observations suggest that the nuclear import of FIH probably involves other proteins, consistent with the hypothesis that its nuclear entry is mediated, at least under these conditions, by association with HIF1α. A classical importin α/β-dependent bipartite nuclear localization signal (NLS) is present at the C-terminus of human HIFα (Depping et al., 2008). As anticipated, siRNA-dependent depletion of importin β1 blocked the nuclear accumulation of HIF1α induced by DMOG treatment (Fig. S3; Fig. 3A, row HIF1α). A lower level of HIF1α protein was detected in DMOG-treated importin β1-depleted cells than in DMOG-treated control cells, suggesting that the nuclear retention of HIF1α somehow reduces its degradation (Fig. S3). Importantly, under the same conditions, DMOG-promoted nuclear entry of FIH was abolished upon importin β1 depletion (Fig. 3A, row FIH). Consistent with this observation, DMOG treatment induced the association of importin β1 with HIF1α and FIH; binding between FIH and importin β1 was HIF1α-dependent, because knockdown of HIF1α abolished the interaction between FIH and importin β1 (Fig. 3B).

**Fig. 4.**
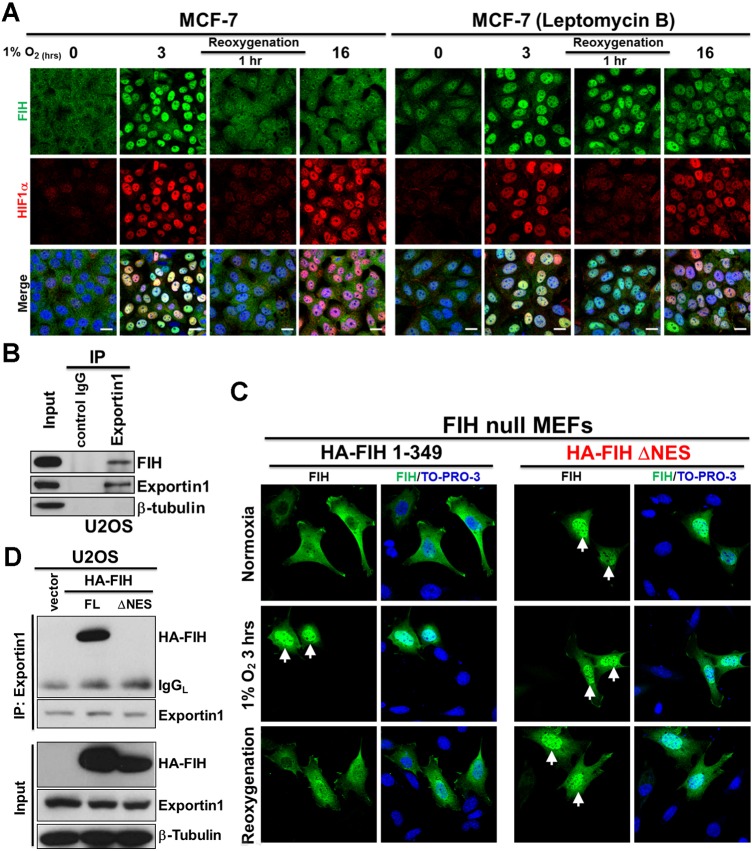
**FIH exits the nucleus via a Leptomycin B-sensitive exportin1-dependent pathway.** (A) Immunofluorescence staining of FIH (green) and HIF1α (red) in MCF7 cells after the indicated hypoxia (0.5% O_2_) and re-oxygenation treatments. TO-PRO-3 (blue) was used to stain nuclei. (B) Total cell lysates from U2OS cells were immunoprecipitated with an anti-exportin1 antibody or control IgG. FIH, exportin1 and β-tubulin levels are indicated. (C) Immunofluorescence staining of FIH (green) in FIH-null mouse embryonic fibroblasts (MEFs) transfected with HA-FIH 1–349 or HA-FIH ΔNES followed by normoxia, hypoxia (1% O_2_, 3 h) or 3 h of hypoxia followed by re-oxygenation for 1 h. TO-PRO-3 (blue) was used to stain nuclei. Arrows indicate nuclear localization of signal. (D) Total cell lysates from U2OS cells transfected with control vector, HA-FIH 1–349 or HA-FIH ΔNES were immunoprecipitated with an anti-exportin 1 antibody. HA-FIH, exportin1 and β-tubulin levels are indicated. FL, full length; IgG_L_, IgG light chain. Scale bars: 20 µm.

We observed that FIH was retained in the nucleus for only a few hours during application of hypoxia (Fig. 1B; Fig. S1C). Interestingly, during re-oxygenation following hypoxia, FIH was observed in the nucleus for up to 1 h, whereas HIF1α was degraded more quickly (Fig. S4A), suggesting that export of nuclear FIH is mediated by an independent process. Nuclear export of proteins is usually mediated by leucine-rich nuclear export signals (NES) that are recognized by nuclear export receptors (Tran et al., 2014). Seven exportins have been described so far, with exportin1 (also referred to as chromosome region maintenance 1, CRM1) being the most abundant (Tran et al., 2014). Leptomycin B is a potent and specific nuclear export inhibitor that inhibits exportin1 (Nishi et al., 1994). We found that Leptomycin B treatment promoted retention of FIH in the nucleus following 16 h of hypoxia and re-oxygenation for 1 h after 3 h of hypoxia, whereas in untreated cells, FIH was no longer detectable in the nucleus under these conditions (Fig. 4A). These results indicate that FIH is exported by a Leptomycin B-sensitive pathway. Consistent with this, endogenous FIH and exportin1 co-immunoprecipitated in U2OS cells (Fig. 4B). Furthermore, a potential NES is predicted within FIH (residues 128–137) using NetNES (http://www.cbs.dtu.dk/services/NetNES/) (Fig. S4B). We therefore constructed a plasmid encoding hemagglutinin (HA)-tagged FIH lacking the predicted NES (HA-FIH ΔNES), and transfected wild-type or ΔNES HA-tagged FIH plasmids into FIH-null mouse embryonic fibroblasts (MEFs). Consistent with the work described above, we found that exogenously expressed wild-type FIH was cytoplasmic, and that nuclear accumulation was observed under hypoxic conditions. Furthermore, after 1 h of re-oxygenation following 3h of hypoxia, FIH was cytoplasmic (Fig. 4C, HA-FIH 1–349 panel), consistent with our observations regarding endogenous FIH (Fig. 4A; Fig. S4A). By contrast, exogenously expressed FIH lacking the predicted NES showed nuclear localization under all conditions (Fig. 4C, HA-FIH ΔNES panel). In addition, exogenously expressed FIH lacking the predicted NES failed to associate with exportin1 (Fig. 4D), highlighting the important role of this sequence in mediating nuclear export.

These data demonstrate that FIH is exported via a Leptomycin B-sensitive exportin1 (CRM1)-dependent pathway. However, under hypoxic conditions, FIH is actively imported by importin β1-mediated nuclear import in association with HIF1α.

## DISCUSSION

FIH was initially isolated as a negatively regulating factor of HIFα following a yeast two-hybrid screen using the final 251 residues of human HIF1α (576–826) as bait (Mahon et al., 2001). The mechanism by which FIH represses HIFα transcriptional activity was not determined in this initial study. Independently, Lando et al. subsequently found that an asparagine residue (N851 in mouse HIF2α, corresponding to N803 in human HIF1α), which is conserved in orthologous vertebrate HIF1α and HIF2α proteins, undergoes hydroxylation (Lando et al., 2002b). They demonstrated that asparaginyl hydroxylation is a mechanism for normoxic repression of transcriptional activation by the HIF1α CAD via blocking the interaction of HIF with the CBP/p300 transcriptional coactivator proteins.

FIH was subsequently shown to be the HIF1α asparaginyl hydroxylase following bioinformatic analyses that predicted FIH to have a tertiary structure that included a modified double-stranded β-helix fold that is typical of the 2-OG-dependent hydroxylases (Hewitson et al., 2002; Lando et al., 2002a). This prediction was subsequently verified by crystallographic analyses (Elkins et al., 2003). Recombinant FIH was shown to catalyse the Fe(II)- and 2-OG-dependent C-3 hydroxylation of an asparagine residue in the CAD of HIFα isoforms (Hewitson et al., 2002; Lando et al., 2002a; McNeill et al., 2002).

FIH-catalysed HIFα hydroxylation blocks the interaction between the transcriptional coactivators/histone acetyl transferases p300/CBP and HIFα (Lando et al., 2002a). *In vitro*, p300 does not bind to HIFα CAD treated with wild-type FIH, but does bind to HIFα CAD treated with a catalytically inactive FIH variant (Hewitson et al., 2002; Lando et al., 2002a). Subsequent work has also revealed that FIH accepts multiple substrates from the ankyrin repeat domain (ARD) family of proteins (Cockman et al., 2006, 2009; Coleman et al., 2007; Janke et al., 2013; Karttunen et al., 2015; Zheng et al., 2008), which are located in different cellular compartments. As a result, intracellular processes that affect the subcellular location of FIH determine its access to different substrates.

Our findings reveal dynamic mechanisms controlling the cellular localization of FIH. We employed both genetic and small-molecule interventions to identify a mechanism by which hypoxia induces nuclear translocation of FIH. Notably, we show that FIH accumulates in the nucleus within a short time window during hypoxia treatment. We provide evidence that the presence of FIH in the nucleus is governed by two opposing processes: nuclear entry by “coupling” of FIH import with HIF1α for importin β1-mediated nuclear import and active export by a Leptomycin B-sensitive exportin1 (CRM1)-mediated nuclear export pathway. We identified a potential NES within FIH (residues 128–137) using bioinformatics and confirmed the results by comparison of NES-deleted FIH and full-length FIH under identical transfection conditions in FIH-null MEFs. We also report that nuclear import of FIH is promoted by inhibition of its own enzyme activity. This is consistent with an earlier report by Cockman et al. showing that the FIH–substrate interactions could be stabilized by pretreatment of cells with the catalytic inhibitor DMOG, most probably as a result of prolongation of otherwise transient interactions between enzyme and substrate (Cockman et al., 2009).

It is also notable that, in addition to the HIF1α isoforms, FIH binds and hydroxylates a diverse array of ARD-containing proteins, including Notch (Coleman et al., 2007; Zheng et al., 2008), apoptosis-stimulating of p53 protein 2 (ASPP2) (Janke et al., 2013) and others (Cockman et al., 2006, 2009; Karttunen et al., 2015). Unlike the very substantial effect of asparaginyl hydroxylation on HIF-mediated transcription, FIH-catalysed ARD hydroxylation has not yet been found to have a clear role in ARD signalling (although it can affect ARD stability) (Kelly et al., 2009). These ARD-containing proteins, however, have a high affinity for FIH and are abundant in cells (Coleman et al., 2007; Wilkins et al., 2009); thus, they compete with HIF1α for asparaginyl hydroxylation, which, as demonstrated in this study, is crucial for facilitating the nuclear import of FIH. In addition, a group of ARD-containing proteins use the RanGDP/AR (RaDAR) complex-mediated system for nuclear import (Lu et al., 2014). Interestingly, the Notch intracellular domain (NICD) has been reported to play a role in the nuclear accumulation of FIH in normoxic cells although the mechanism has not been explored (Zheng et al., 2008). Our findings may enable new insights into the paradox that FIH effectively hydroxylates HIF1α despite the presence of numerous and abundant competing ARD substrates. Competition between ARDs and HIFα for binding to FIH might not only directly regulate FIH-catalysed HIFα hydroxylation (as previously proposed) (Cockman et al., 2006; Schmierer et al., 2010), but also regulate the cytoplasmic versus nuclear localizations of FIH. The new data suggest a more complex and dynamic interface with the HIF transcriptional response, where FIH might be particularly important in modulating transitions between normoxic and hypoxic states. It is also important to note that FIH is a dimer (Elkins et al., 2003) and thus has the potential to bind more than one protein (substrate) simultaneously, potentially enabling further fine-tuning of the role of FIH in the hypoxic response.

The results shown in this study could explain why several earlier studies failed to detect nuclear FIH under hypoxia (Linke et al., 2004; Metzen et al., 2003; Soilleux et al., 2005; Stolze et al., 2004), because the narrow window of the nuclear impact of FIH might have been missed. The results also potentially explain why FIH has sometimes been observed in the nucleus in pathological tissues (Kroeze et al., 2010; Tan et al., 2007), despite being largely a cytoplasmic protein in normoxic cells. A detailed understanding of the biological importance of cytoplasmic and nuclear FIH function could help to clarify whether nuclear FIH causes or simply associates with pathological conditions.

## MATERIALS AND METHODS

### Cell culture, reagents and transfections

U2OS, HKe3, MCF7 cells and FIH-null MEFs were cultured in DMEM supplemented with 10% fetal bovine serum (Invitrogen) and antibiotics. All cells were kept at 37°C and under 10% CO_2_. No mycoplasma contamination was detected in the cell lines used. Hypoxic incubations were performed using InvivO2 400 hypoxic workstations (Ruskinn Technologies, Bridgend, UK). Chemicals DMOG, IOX1, IOX2, FG2216, VGB10B (IOX4), DFO and DM-NOFD were obtained or synthesized as reported (Chan et al., 2015, 2016; Chowdhury et al., 2013; Hopkinson et al., 2013; McDonough et al., 2005; Mole et al., 2003; Yeh et al., 2017).

siRNA oligos against the genes encoding for FIH (MU-004073-02-0002), HIF1α (MU-004018-05-0002), HIF2α (MU-004814-01-0002), HIF1β (MU-007207-01-0002) and importin β1 (MU-017523-01-0002) were purchased from Dharmacon. Sequences are available from Dharmacon, or on request. As negative control, we used siGENOME RISC-Free siRNA (Dharmacon). Cells were transfected with the indicated siRNA oligos at a final concentration of 35 nM using DharmaFECT 1 reagent (Dharmacon).

Wild-type HA-tagged FIH (HA-FIH 1–349) plasmid was cloned as described previously (Coleman et al., 2007). HA-tagged FIH plasmid lacking the predicted NES 128–137 (HA-FIH ΔNES) was cloned by site-directed mutagenesis. Transfections were performed with FuGENE 6 (Promega), according to the manufacturer's instructions.

### Western blot analysis

Western blot analysis was carried out with lysates from cells using urea buffer (8 M urea, 1 M thiourea, 0.5% CHAPS, 50 mM dithiothreitol and 24 mM spermine). For preparation of cytoplasmic and nuclear proteins, NE-PER nuclear and cytoplasmic extraction reagents (Pierce) were used in accordance with the manufacturer's protocol. Cytoplasmic and nuclear fractions were isolated from U2OS cells after the indicated treatments. β-tubulin was used as a loading control for the cytoplasmic fraction, whereas PARP was used as a loading control for the nuclear fraction. For immunoprecipitations, the cells were lysed for 30 min at 4°C in pNAS buffer (50 mM Tris-HCl at pH 7.5, 120 mM NaCl, 1 mM EDTA and 0.1% Nonidet P-40) containing protease inhibitors. Cell extracts were then precleared with protein G beads and incubated with antibodies against importin β1 (2 μl/mg protein lysate; Cell Signaling Technology, 8673, rabbit polyclonal), exportin1 (2 μg/mg protein lysate; Sigma-Aldrich, E7784, rabbit polyclonal), HIF1α (2 μg/mg protein lysate; Novus Biologicals, NB100-479, rabbit polyclonal) or p300 (2 μg/mg protein lysate, Millipore, 05-257, mouse monoclonal RW128) for 16 h at 4°C. Immunoprecipitates were washed four times with cold PBS followed by the addition of SDS sample buffer. The bound proteins were separated on SDS–polyacrylamide gels and subjected to immunoblotting with the indicated antibodies.

Primary antibodies were from Novus Biologicals (HIF1α, 1:1000, NB100-479, rabbit polyclonal), Abcam (β-tubulin, 1:5000, ab6046, rabbit polyclonal; GAPDH, 1:2000, ab9385, rabbit polyclonal; Ku80, 1:2000, ab80592, rabbit monoclonal EPR3468), Sigma-Aldrich (exportin1, 1:1000, E7784, rabbit polyclonal), BD Transduction Laboratories (HIF1α, 1:1000, 610958, mouse monoclonal clone 54/HIF1α), Cell Signaling Technology (PARP, 1:1000, 9542, rabbit polyclonal; HIF1β, 1:1000, 5537, rabbit monoclonal D28F3; importin β1, 1:1000, 8673, rabbit polyclonal), Santa Cruz Biotechnology (HA, 1:1000, sc-7392, mouse monoclonal clone F-7), HIF2α (1:50, clone 190b) (Wiesener et al., 1998), HIF1α hydroxy-Asn^803^ (N803OH) (1:5000, mouse monoclonal) (Lee et al., 2008) and FIH (1:200, mouse monoclonal 162C) (Stolze et al., 2004). Signals were detected using an ECL detection system (GE Healthcare) and evaluated by ImageJ 1.42q software (National Institutes of Health).

### Immunofluorescence microscopy

Cells were fixed in 4% PBS–paraformaldehyde for 15 min, incubated in 0.1% Triton X-100 for 5 min on ice, then in 0.2% fish skin gelatin in PBS for 1 h and stained for 1 h with an anti-FIH antibody (1:50, mouse monoclonal 162C) (Stolze et al., 2004), anti-HIF1α N803OH (1:500, mouse monoclonal) (Lee et al., 2008) and anti-HIF1α antibody (1:100, BD Transduction Laboratories 610958, mouse monoclonal clone 54/HIF1α or 1:100, Novus Biologicals NB100-479, rabbit polyclonal). Protein expression was detected using Alexa Fluor 488 or 546 (1:400, Molecular Probes) for 20 min. DAPI or TO-PRO-3 (Invitrogen) was used to stain nucleic acids (1:1000). Samples were observed using a confocal microscope system (LSM 510 or LSM 710; Carl Zeiss). Acquired images were analysed by ImageJ 1.42q software (National Institutes of Health) using in-house plugins written for quantification of nuclear signal. Four high-power fields were selected for analysis of each treatment. To avoid being biased by the FIH staining, each field was selected by viewing nuclear (DAPI) staining only to identify near-confluent cells and thereby maximise the cell numbers (∼100 cells) included in the analysis. For each high-power field, binary image masks were created of FIH- and DAPI-positive staining to define regions of interest (ROI) for analysis. The DAPI staining mask was used to define the nuclear ROI, which was then applied, by the image calculator, to the original FIH staining images to isolate nuclear staining within each image. Using the image calculator, the DAPI mask was subtracted from the FIH mask to create a staining mask defining the non-nuclear ROI. Quantitative fluorescence data were exported from ImageJ-generated histograms into Microsoft Excel software for further analysis and presentation. Cells with the ratio of nuclear FIH mean intensity over non-nuclear FIH mean intensity greater than 2 were considered nuclear FIH-positive.

### Statistical analysis and repeatability of experiments

Each experiment was repeated at least twice. Unless otherwise noted, data are presented as mean±s.d. A two-tailed, unpaired Student's *t*-test was used to compare two groups of independent samples. *P*<0.05 was considered statistically significant.

## Supplementary Material

Supplementary information

## References

[JCS219782C1] Adams R. L. and Wente S. R. (2013). Uncovering nuclear pore complexity with innovation. *Cell* 152, 1218-1221. 10.1016/j.cell.2013.02.042 23498931 PMC3672239

[JCS219782C2] Aitchison J. D. and Rout M. P. (2012). The yeast nuclear pore complex and transport through it. *Genetics* 190, 855-883. 10.1534/genetics.111.127803 22419078 PMC3296253

[JCS219782C3] Berra E., Benizri E., Ginouves A., Volmat V., Roux D. and Pouyssegur J. (2003). HIF prolyl-hydroxylase 2 is the key oxygen sensor setting low steady-state levels of HIF-1alpha in normoxia. *EMBO J.* 22, 4082-4090. 10.1093/emboj/cdg392 12912907 PMC175782

[JCS219782C4] Bracken C. P., Fedele A. O., Linke S., Balrak W., Lisy K., Whitelaw M. L. and Peet D. J. (2006). Cell-specific regulation of hypoxia-inducible factor (HIF)-1alpha and HIF-2alpha stabilization and transactivation in a graded oxygen environment. *J. Biol. Chem.* 281, 22575-22585. 10.1074/jbc.M600288200 16760477

[JCS219782C5] Chan M. C., Atasoylu O., Hodson E., Tumber A., Leung I. K., Chowdhury R., Gomez-Perez V., Demetriades M., Rydzik A. M., Holt-Martyn J. et al. (2015). Potent and selective triazole-based inhibitors of the hypoxia-inducible factor prolyl-hydroxylases with activity in the murine brain. *PLoS ONE* 10, e0132004 10.1371/journal.pone.0132004 26147748 PMC4492579

[JCS219782C6] Chan M. C., Ilott N. E., Schodel J., Sims D., Tumber A., Lippl K., Mole D. R., Pugh C. W., Ratcliffe P. J., Ponting C. P. et al. (2016). Tuning the transcriptional response to hypoxia by inhibiting hypoxia-inducible factor (HIF) Prolyl and asparaginyl hydroxylases. *J. Biol. Chem.* 291, 20661-20673. 10.1074/jbc.M116.749291 27502280 PMC5034057

[JCS219782C7] Chilov D., Camenisch G., Kvietikova I., Ziegler U., Gassmann M. and Wenger R. H. (1999). Induction and nuclear translocation of hypoxia-inducible factor-1 (HIF-1): heterodimerization with ARNT is not necessary for nuclear accumulation of HIF-1alpha. *J. Cell Sci.* 112, 1203-1212. 10085255 10.1242/jcs.112.8.1203

[JCS219782C8] Chowdhury R., Candela-Lena J. I., Chan M. C., Greenald D. J., Yeoh K. K., Tian Y.-M., McDonough M. A., Tumber A., Rose N. R., Conejo-Garcia A. et al. (2013). Selective small molecule probes for the hypoxia inducible factor (HIF) prolyl hydroxylases. *ACS Chem. Biol.* 8, 1488-1496. 10.1021/cb400088q 23683440

[JCS219782C9] Cockman M. E., Lancaster D. E., Stolze I. P., Hewitson K. S., McDonough M. A., Coleman M. L., Coles C. H., Yu X., Hay R. T., Ley S. C. et al. (2006). Posttranslational hydroxylation of ankyrin repeats in IkappaB proteins by the hypoxia-inducible factor (HIF) asparaginyl hydroxylase, factor inhibiting HIF (FIH). *Proc. Natl. Acad. Sci. USA* 103, 14767-14772. 10.1073/pnas.0606877103 17003112 PMC1578504

[JCS219782C10] Cockman M. E., Webb J. D., Kramer H. B., Kessler B. M. and Ratcliffe P. J. (2009). Proteomics-based identification of novel factor inhibiting hypoxia-inducible factor (FIH) substrates indicates widespread asparaginyl hydroxylation of ankyrin repeat domain-containing proteins. *Mol. Cell. Proteomics* 8, 535-546. 10.1074/mcp.M800340-MCP200 18936059 PMC2649815

[JCS219782C11] Coleman M. L., McDonough M. A., Hewitson K. S., Coles C., Mecinovic J., Edelmann M., Cook K. M., Cockman M. E., Lancaster D. E., Kessler B. M. et al. (2007). Asparaginyl hydroxylation of the Notch ankyrin repeat domain by factor inhibiting hypoxia-inducible factor. *J. Biol. Chem.* 282, 24027-24038. 10.1074/jbc.M704102200 17573339

[JCS219782C12] Dann C. E. III, Bruick R. K. and Deisenhofer J. (2002). Structure of factor-inhibiting hypoxia-inducible factor 1: An asparaginyl hydroxylase involved in the hypoxic response pathway. *Proc. Natl. Acad. Sci. USA* 99, 15351-15356. 10.1073/pnas.202614999 12432100 PMC137720

[JCS219782C13] Depping R., Steinhoff A., Schindler S. G., Friedrich B., Fagerlund R., Metzen E., Hartmann E. and Köhler M. (2008). Nuclear translocation of hypoxia-inducible factors (HIFs): involvement of the classical importin alpha/beta pathway. *Biochim. Biophys. Acta* 1783, 394-404. 10.1016/j.bbamcr.2007.12.006 18187047

[JCS219782C14] Depping R., Jelkmann W. and Kosyna F. K. (2015). Nuclear-cytoplasmatic shuttling of proteins in control of cellular oxygen sensing. *J. Mol. Med. (Berl.)* 93, 599-608. 10.1007/s00109-015-1276-0 25809665

[JCS219782C15] Elkins J. M., Hewitson K. S., McNeill L. A., Seibel J. F., Schlemminger I., Pugh C. W., Ratcliffe P. J. and Schofield C. J. (2003). Structure of factor-inhibiting hypoxia-inducible factor (HIF) reveals mechanism of oxidative modification of HIF-1 alpha. *J. Biol. Chem.* 278, 1802-1806. 10.1074/jbc.C200644200 12446723

[JCS219782C16] Epstein A. C., Gleadle J. M., McNeill L. A., Hewitson K. S., O'Rourke J., Mole D. R., Mukherji M., Metzen E., Wilson M. I., Dhanda A. et al. (2001). C. elegans EGL-9 and mammalian homologs define a family of dioxygenases that regulate HIF by prolyl hydroxylation. *Cell* 107, 43-54. 10.1016/S0092-8674(01)00507-4 11595184

[JCS219782C17] Hewitson K. S., McNeill L. A., Riordan M. V., Tian Y.-M., Bullock A. N., Welford R. W., Elkins J. M., Oldham N. J., Bhattacharya S., Gleadle J. M. et al. (2002). Hypoxia-inducible factor (HIF) asparagine hydroxylase is identical to factor inhibiting HIF (FIH) and is related to the cupin structural family. *J. Biol. Chem.* 277, 26351-26355. 10.1074/jbc.C200273200 12042299

[JCS219782C18] Hopkinson R. J., Tumber A., Yapp C., Chowdhury R., Aik W., Che K. H., Li X. S., Kristensen J. B. L., King O. N. F., Chan M. C. et al. (2013). 5-Carboxy-8-hydroxyquinoline is a broad spectrum 2-oxoglutarate oxygenase inhibitor which causes iron translocation. *Chem. Sci.* 4, 3110-3117. 10.1039/c3sc51122g 26682036 PMC4678600

[JCS219782C19] Janke K., Brockmeier U., Kuhlmann K., Eisenacher M., Nolde J., Meyer H. E., Mairbaurl H. and Metzen E. (2013). Factor inhibiting HIF-1 (FIH-1) modulates protein interactions of apoptosis-stimulating p53 binding protein 2 (ASPP2). *J. Cell Sci.* 126, 2629-2640. 10.1242/jcs.117564 23606740

[JCS219782C20] Kaelin W. G. Jr. and Ratcliffe P. J. (2008). Oxygen sensing by metazoans: the central role of the HIF hydroxylase pathway. *Mol. Cell* 30, 393-402. 10.1016/j.molcel.2008.04.009 18498744

[JCS219782C21] Kang J., Shin S. H., Yoon H., Huh J., Shin H. W., Chun Y. S. and Park J. W. (2017). FIH is an oxygen sensor in ovarian cancer for G9a/GLP-driven epigenetic regulation of metastasis-related genes. *Cancer Res.* 78, 1184-1199. 10.1158/0008-5472.CAN-17-2506 29259012

[JCS219782C22] Karttunen S., Duffield M., Scrimgeour N. R., Squires L., Lim W. L., Dallas M. L., Scragg J. L., Chicher J., Dave K. A., Whitelaw M. L. et al. (2015). Oxygen-dependent hydroxylation by FIH regulates the TRPV3 ion channel. *J. Cell Sci.* 128, 225-231. 10.1242/jcs.158451 25413349

[JCS219782C23] Kelly L., McDonough M. A., Coleman M. L., Ratcliffe P. J. and Schofield C. J. (2009). Asparagine beta-hydroxylation stabilizes the ankyrin repeat domain fold. *Mol. Biosyst.* 5, 52-58. 10.1039/B815271C 19081931

[JCS219782C24] Kiriakidis S., Henze A.-T., Kruszynska-Ziaja I., Skobridis K., Theodorou V., Paleolog E. M. and Mazzone M. (2015). Factor-inhibiting HIF-1 (FIH-1) is required for human vascular endothelial cell survival. *FASEB J.* 29, 2814-2827. 10.1096/fj.14-252379 25837583

[JCS219782C25] Kroeze S. G., Vermaat J. S., van Brussel A., van Melick H. H. E., Voest E. E., Jonges T. G. N., van Diest P. J., Hinrichs J., Bosch J. L. H. R. and Jans J. J. (2010). Expression of nuclear FIH independently predicts overall survival of clear cell renal cell carcinoma patients. *Eur. J. Cancer* 46, 3375-3382. 10.1016/j.ejca.2010.07.018 20709525

[JCS219782C26] Kuzmanov A., Wielockx B., Rezaei M., Kettelhake A. and Breier G. (2012). Overexpression of factor inhibiting HIF-1 enhances vessel maturation and tumor growth via platelet-derived growth factor-C. *Int. J. Cancer* 131, E603-E613. 10.1002/ijc.27360 22095574

[JCS219782C27] Lancaster D. E., McNeill L. A., McDonough M. A., Aplin R. T., Hewitson K. S., Pugh C. W., Ratcliffe P. J. and Schofield C. J. (2004). Disruption of dimerization and substrate phosphorylation inhibit factor inhibiting hypoxia-inducible factor (FIH) activity. *Biochem. J.* 383, 429-437. 10.1042/BJ20040735 15239670 PMC1133735

[JCS219782C28] Lando D., Peet D. J., Gorman J. J., Whelan D. A., Whitelaw M. L. and Bruick R. K. (2002a). FIH-1 is an asparaginyl hydroxylase enzyme that regulates the transcriptional activity of hypoxia-inducible factor. *Genes Dev.* 16, 1466-1471. 10.1101/gad.991402 12080085 PMC186346

[JCS219782C29] Lando D., Peet D. J., Whelan D. A., Gorman J. J. and Whitelaw M. L. (2002b). Asparagine hydroxylation of the HIF transactivation domain a hypoxic switch. *Science* 295, 858-861. 10.1126/science.1068592 11823643

[JCS219782C30] Lee S.-H., Jeong Hee M., Eun Ah C., Ryu S.-E. and Myung Kyu L. (2008). Monoclonal antibody-based screening assay for factor inhibiting hypoxia-inducible factor inhibitors. *J. Biomol. Screen.* 13, 494-503. 10.1177/1087057108318800 18566480

[JCS219782C31] Liang K., Ding X.-Q., Lin C. and Kang Y. J. (2015). Featured Article: Hypoxia-inducible factor-1alpha dependent nuclear entry of factor inhibiting HIF-1. *Exp. Biol. Med. (Maywood)* 240, 1446-1451. 10.1177/1535370215570821 25687434 PMC4935306

[JCS219782C32] Linke S., Stojkoski C., Kewley R. J., Booker G. W., Whitelaw M. L. and Peet D. J. (2004). Substrate requirements of the oxygen-sensing asparaginyl hydroxylase factor-inhibiting hypoxia-inducible factor. *J. Biol. Chem.* 279, 14391-14397. 10.1074/jbc.M313614200 14734545

[JCS219782C33] Lu M., Zak J., Chen S., Sanchez-Pulido L., Severson D. T., Endicott J., Ponting C. P., Schofield C. J. and Lu X. (2014). A code for RanGDP binding in ankyrin repeats defines a nuclear import pathway. *Cell* 157, 1130-1145. 10.1016/j.cell.2014.05.006 24855949

[JCS219782C34] Mahon P. C., Hirota K. and Semenza G. L. (2001). FIH-1: a novel protein that interacts with HIF-1alpha and VHL to mediate repression of HIF-1 transcriptional activity. *Genes Dev.* 15, 2675-2686. 10.1101/gad.924501 11641274 PMC312814

[JCS219782C35] Marxsen J. H., Stengel P., Doege K., Heikkinen P., Jokilehto T., Wagner T., Jelkmann W., Jaakkola P. and Metzen E. (2004). Hypoxia-inducible factor-1 (HIF-1) promotes its degradation by induction of HIF-alpha-prolyl-4-hydroxylases. *Biochem. J.* 381, 761-767. 10.1042/BJ20040620 15104534 PMC1133886

[JCS219782C36] McDonough M. A., McNeill L. A., Tilliet M., Papamicaël C. A., Chen Q.-Y., Banerji B., Hewitson K. S. and Schofield C. J. (2005). Selective inhibition of factor inhibiting hypoxia-inducible factor. *J. Am. Chem. Soc.* 127, 7680-7681. 10.1021/ja050841b 15913349

[JCS219782C37] McNeill L. A., Hewitson K. S., Claridge T. D., Seibel J. F., Horsfall L. E. and Schofield C. J. (2002). Hypoxia-inducible factor asparaginyl hydroxylase (FIH-1) catalyses hydroxylation at the beta-carbon of asparagine-803. *Biochem. J.* 367, 571-575. 10.1042/bj20021162 12215170 PMC1222951

[JCS219782C38] Metzen E., Berchner-Pfannschmidt U., Stengel P., Marxsen J. H., Stolze I., Klinger M., Huang W. Q., Wotzlaw C., Hellwig-Burgel T., Jelkmann W. et al. (2003). Intracellular localisation of human HIF-1 alpha hydroxylases: implications for oxygen sensing. *J. Cell Sci.* 116, 1319-1326. 10.1242/jcs.00318 12615973

[JCS219782C39] Mole D. R., Schlemminger I., McNeill L. A., Hewitson K. S., Pugh C. W., Ratcliffe P. J. and Schofield C. J. (2003). 2-oxoglutarate analogue inhibitors of HIF prolyl hydroxylase. *Bioorg. Med. Chem. Lett.* 13, 2677-2680. 10.1016/S0960-894X(03)00539-0 12873492

[JCS219782C40] Myllyharju J. (2013). Prolyl 4-hydroxylases, master regulators of the hypoxia response. *Acta Physiol. (Oxf.)* 208, 148-165. 10.1111/apha.12096 23489300

[JCS219782C41] Nishi K., Yoshida M., Fujiwara D., Nishikawa M., Horinouchi S. and Beppu T. (1994). Leptomycin B targets a regulatory cascade of crm1, a fission yeast nuclear protein, involved in control of higher order chromosome structure and gene expression. *J. Biol. Chem.* 269, 6320-6324. 8119981

[JCS219782C42] Pelletier J., Dayan F., Durivault J., Ilc K., Pécou E., Pouysségur J. and Mazure N. M. (2012). The asparaginyl hydroxylase factor-inhibiting HIF is essential for tumor growth through suppression of the p53-p21 axis. *Oncogene* 31, 2989-3001. 10.1038/onc.2011.471 22002313

[JCS219782C43] Peng H., Hamanaka R. B., Katsnelson J., Hao L.-L., Yang W., Chandel N. S. and Lavker R. M. (2012a). MicroRNA-31 targets FIH-1 to positively regulate corneal epithelial glycogen metabolism. *FASEB J.* 26, 3140-3147. 10.1096/fj.11-198515 22532441 PMC3405266

[JCS219782C44] Peng H., Kaplan N., Hamanaka R. B., Katsnelson J., Blatt H., Yang W., Hao L., Bryar P. J., Johnson R. S., Getsios S. et al. (2012b). microRNA-31/factor-inhibiting hypoxia-inducible factor 1 nexus regulates keratinocyte differentiation. *Proc. Natl. Acad. Sci. USA* 109, 14030-14034. 10.1073/pnas.1111292109 22891326 PMC3435188

[JCS219782C45] Ratcliffe P. J. (2007). HIF-1 and HIF-2: working alone or together in hypoxia? *J. Clin. Invest.* 117, 862-865. 10.1172/JCI31750 17404612 PMC1838952

[JCS219782C46] Ratcliffe P. J. (2013). Oxygen sensing and hypoxia signalling pathways in animals: the implications of physiology for cancer. *J. Physiol.* 591, 2027-2042. 10.1113/jphysiol.2013.251470 23401619 PMC3634517

[JCS219782C47] Rodriguez J., Pilkington R., Garcia Munoz A., Nguyen L. K., Rauch N., Kennedy S., Monsefi N., Herrero A., Taylor C. T. and von Kriegsheim A. (2016). Substrate-trapped interactors of PHD3 and FIH cluster in distinct signaling pathways. *Cell Rep* 14, 2745-2760. 10.1016/j.celrep.2016.02.043 26972000 PMC4805855

[JCS219782C48] Schmierer B., Novák B. and Schofield C. J. (2010). Hypoxia-dependent sequestration of an oxygen sensor by a widespread structural motif can shape the hypoxic response--a predictive kinetic model. *BMC Syst. Biol.* 4, 139 10.1186/1752-0509-4-139 20955552 PMC2984394

[JCS219782C49] Scholz C. C., Rodriguez J., Pickel C., Burr S., Fabrizio J.-A., Nolan K. A., Spielmann P., Cavadas M. A. S., Crifo B., Halligan D. N. et al. (2016). FIH regulates cellular metabolism through hydroxylation of the deubiquitinase OTUB1. *PLoS Biol.* 14, e1002347 10.1371/journal.pbio.1002347 26752685 PMC4709136

[JCS219782C50] Sim J., Cowburn A. S., Palazon A., Madhu B., Tyrakis P. A., Macias D., Bargiela D. M., Pietsch S., Gralla M., Evans C. E. et al. (2018). The factor inhibiting HIF asparaginyl hydroxylase regulates oxidative metabolism and accelerates metabolic adaptation to hypoxia. *Cell Metab.* 27, 898-913 e7. 10.1016/j.cmet.2018.02.020 29617647 PMC5887987

[JCS219782C51] Soilleux E. J., Turley H., Tian Y. M., Pugh C. W., Gatter K. C. and Harris A. L. (2005). Use of novel monoclonal antibodies to determine the expression and distribution of the hypoxia regulatory factors PHD-1, PHD-2, PHD-3 and FIH in normal and neoplastic human tissues. *Histopathology* 47, 602-610. 10.1111/j.1365-2559.2005.02280.x 16324198

[JCS219782C52] Stolze I. P., Tian Y.-M., Appelhoff R. J., Turley H., Wykoff C. C., Gleadle J. M. and Ratcliffe P. J. (2004). Genetic analysis of the role of the asparaginyl hydroxylase factor inhibiting hypoxia-inducible factor (FIH) in regulating hypoxia-inducible factor (HIF) transcriptional target genes [corrected]. *J. Biol. Chem.* 279, 42719-42725. 10.1074/jbc.M406713200 15302861

[JCS219782C53] Tan E. Y., Campo L., Han C., Turley H., Pezzella F., Gatter K. C., Harris A. L. and Fox S. B. (2007). Cytoplasmic location of factor-inhibiting hypoxia-inducible factor is associated with an enhanced hypoxic response and a shorter survival in invasive breast cancer. *Breast Cancer Res.* 9, R89 10.1186/bcr1838 18096060 PMC2246192

[JCS219782C54] Tian Y.-M., Yeoh K. K., Lee M. K., Eriksson T., Kessler B. M., Kramer H. B., Edelmann M. J., Willam C., Pugh C. W., Schofield C. J. et al. (2011). Differential sensitivity of hypoxia inducible factor hydroxylation sites to hypoxia and hydroxylase inhibitors. *J. Biol. Chem.* 286, 13041-13051. 10.1074/jbc.M110.211110 21335549 PMC3075650

[JCS219782C55] Tran E. J., King M. C. and Corbett A. H. (2014). Macromolecular transport between the nucleus and the cytoplasm: advances in mechanism and emerging links to disease. *Biochim. Biophys. Acta* 1843, 2784-2795. 10.1016/j.bbamcr.2014.08.003 25116306 PMC4161953

[JCS219782C56] Wang Y., Ngo V. N., Marani M., Yang Y., Wright G., Staudt L. M. and Downward J. (2010). Critical role for transcriptional repressor Snail2 in transformation by oncogenic RAS in colorectal carcinoma cells. *Oncogene* 29, 4658-4670. 10.1038/onc.2010.218 20562906 PMC7646260

[JCS219782C57] Wang Y., Bu F., Royer C., Serres S., Larkin J. R., Soto M. S., Sibson N. R., Salter V., Fritzsche F., Turnquist C. et al. (2014). ASPP2 controls epithelial plasticity and inhibits metastasis through beta-catenin-dependent regulation of ZEB1. *Nat. Cell Biol.* 16, 1092-1104. 10.1038/ncb3050 25344754

[JCS219782C58] Wiesener M. S., Turley H., Allen W. E., Willam C., Eckardt K. U., Talks K. L., Wood S. M., Gatter K. C., Harris A. L., Pugh C. W. et al. (1998). Induction of endothelial PAS domain protein-1 by hypoxia: characterization and comparison with hypoxia-inducible factor-1alpha. *Blood* 92, 2260-2268. 9746763

[JCS219782C59] Wilkins S. E., Hyvärinen J., Chicher J., Gorman J. J., Peet D. J., Bilton R. L. and Koivunen P. (2009). Differences in hydroxylation and binding of Notch and HIF-1alpha demonstrate substrate selectivity for factor inhibiting HIF-1 (FIH-1). *Int. J. Biochem. Cell Biol.* 41, 1563-1571. 10.1016/j.biocel.2009.01.005 19401150

[JCS219782C60] Yeh T.-L., Leissing T. M., Abboud M. I., Thinnes C. C., Atasoylu O., Holt-Martyn J. P., Zhang D., Tumber A., Lippl K., Lohans C. T. et al. (2017). Molecular and cellular mechanisms of HIF prolyl hydroxylase inhibitors in clinical trials. *Chem. Sci.* 8, 7651-7668. 10.1039/C7SC02103H 29435217 PMC5802278

[JCS219782C61] Zhang N., Fu Z., Linke S., Chicher J., Gorman J. J., Visk D., Haddad G. G., Poellinger L., Peet D. J., Powell F. et al. (2010). The asparaginyl hydroxylase factor inhibiting HIF-1alpha is an essential regulator of metabolism. *Cell Metab.* 11, 364-378. 10.1016/j.cmet.2010.03.001 20399150 PMC2893150

[JCS219782C62] Zheng X., Linke S., Dias J. M., Gradin K., Wallis T. P., Hamilton B. R., Gustafsson M., Ruas J. L., Wilkins S., Bilton R. L. et al. (2008). Interaction with factor inhibiting HIF-1 defines an additional mode of cross-coupling between the Notch and hypoxia signaling pathways. *Proc. Natl. Acad. Sci. USA* 105, 3368-3373. 10.1073/pnas.0711591105 18299578 PMC2265116

