# Supplementary material for: Nuclear entry and export of FIH are mediated by HIF1α and exportin1, respectively
Source: J Cell Sci. 2018 Nov 19;131(22):jcs219782. doi: 10.1242/jcs.219782 (PMC6250434; doi:10.1242/jcs.219782)
Supplement: Supplementary information [file joces-131-219782-s1.pdf]

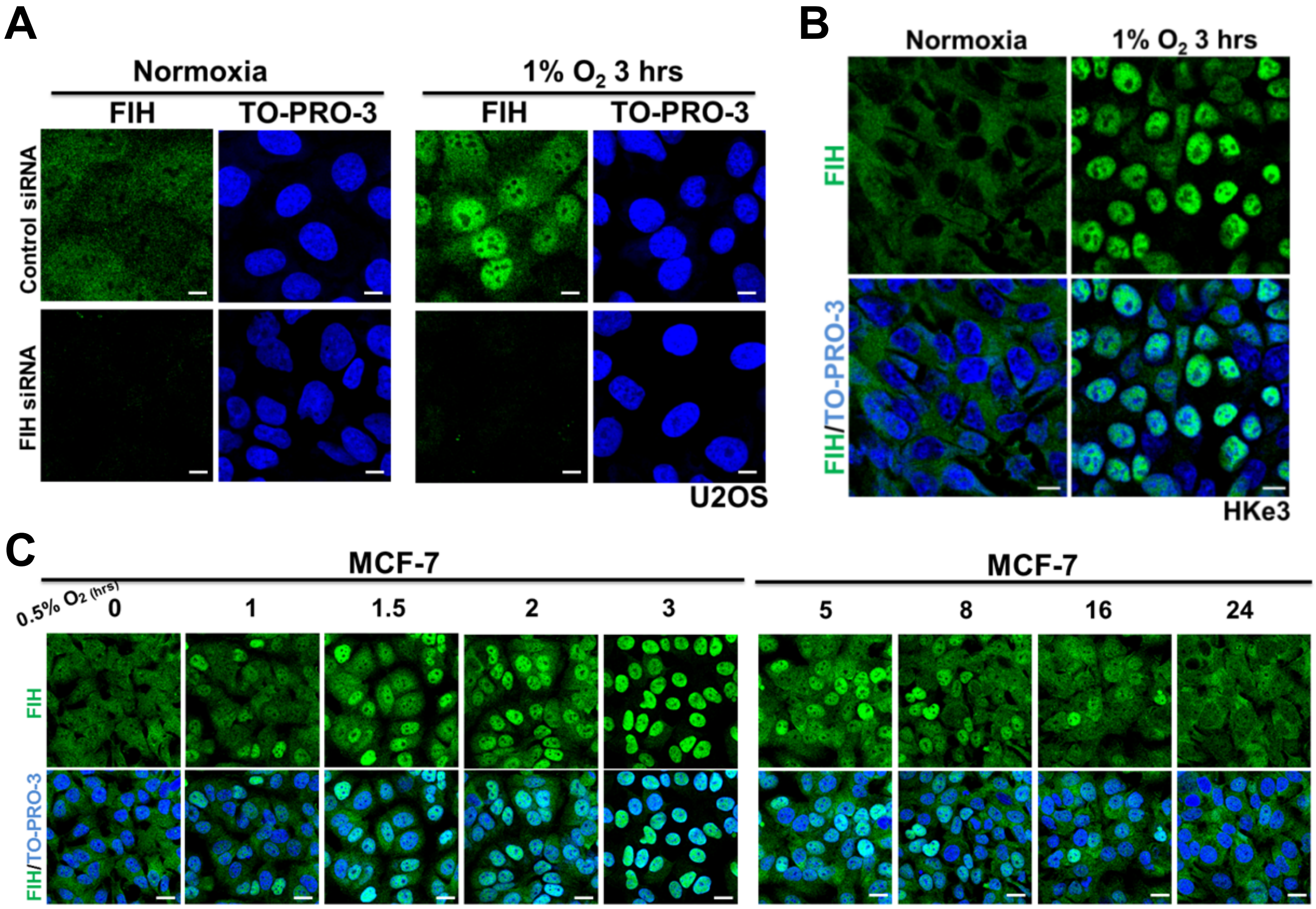

Supplementary Figure 1 – Evidence that hypoxia induces nuclear entry of FIH.

(*A*) Immunofluorescence staining of FIH (green) in U2OS cells with the indicated treatments. U2OS cells were transfected with control siRNA or FIH siRNA for 3 days, followed by cultured in normoxia (20 % O_2) or 3 hours' hypoxia (1 % O_2). TO-PRO-3 (blue) was used to stain nuclei. Scale bar: 10 µm.

(*B*) Immunofluorescence staining of FIH (green) in HKe3 cells cultured in normoxia (20 % O_2) or 3 hours hypoxia (1 % O_2) treatment. TO-PRO-3 (blue) was used to stain nuclei. Scale bar: 10 µm.

(*C*) Immunofluorescence staining of FIH (green) in MCF7 cells in hypoxia (0.5% O_2) at the indicated time points. TO-PRO-3 (blue) was used to stain nuclei. Scale bar: 20 µm.

Journal of Cell Science • Supplementary information

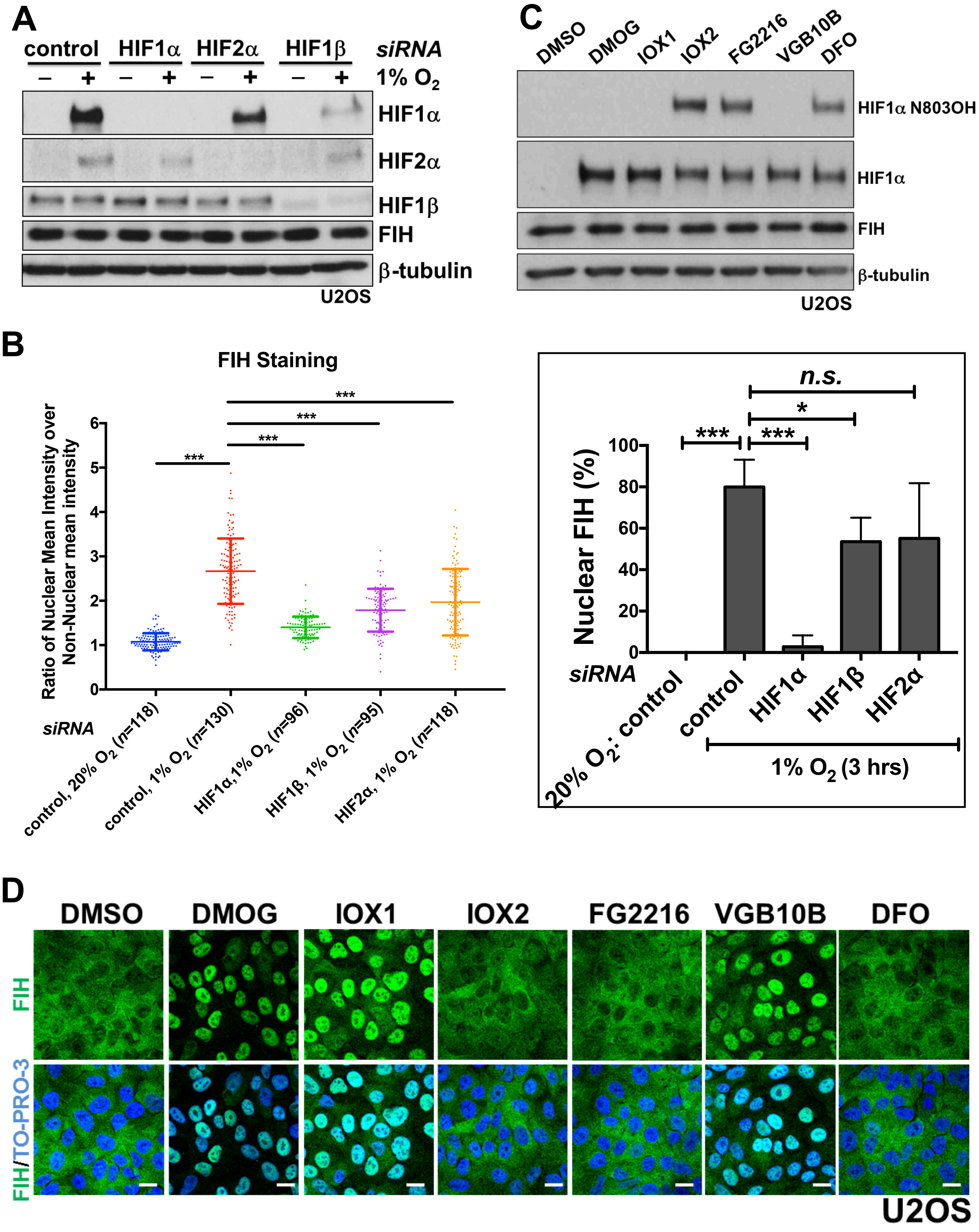
A
control
HIF1α
HIF2α
HIF1β
siRNA
1% O2
HIF1α
HIF2α
HIF1β
FIH
β-tubulin
U2OS
B
FIH Staining
Ratio of Nuclear Mean Intensity over Non-Nuclear mean intensity
siRNA
control, 20% O2 (n=118)
control, 1% O2 (n=130)
HIF1α,1% O2 (n=96)
HIF1β, 1% O2 (n=95)
HIF2α, 1% O2 (n=118)
C
DMSO
DMOG
IOX1
IOX2
FG2216
VGB10B
DFO
HIF1α N803OH
HIF1α
FIH
β-tubulin
U2OS
n.s.
Nuclear FIH (%)
siRNA
20% O2: control
control
HIF1α
HIF1β
HIF2α
1% O2 (3 hrs)
D
DMSO
DMOG
IOX1
IOX2
FG2216
VGB10B
DFO
FIH
FIH/TO-PRO-3
U2OS

Supplementary Figure 2 - Nuclear entry of FIH is mainly HIF1α-dependent and is regulated by inhibition of FIH enzyme activity.

(*A*) Protein levels of HIF1α, HIF2α, HIF1β and FIH in U2OS cells with the indicated treatments. U2OS cells were transfected with the indicated siRNA for 3 days, followed by culture in normoxia (20 % O_2) or 3 hours hypoxia (1 % O_2) treatment. β-tubulin was used as a loading control.

(*B*) The graphs show the ratio of nuclear FIH mean intensity over non-nuclear FIH mean intensity (left) and the percentage of nuclear FIH positive cells (right) with the indicated treatment evaluated by ImageJ. Left: *n* represents the total number of cells evaluated by ImageJ over 4 random fields. Each dot in the plot represents the ratio of nuclear FIH mean intensity over non-nuclear FIH mean intensity in an individual cell. Right: Cells with the ratio of nuclear FIH mean intensity over non-nuclear FIH mean intensity bigger than 2 are considered as nuclear FIH positive (n = 4 random fields). Data are mean ± s.d. * $P < 0.05$. *** $P < 0.001$. *n.s.* $P > 0.05$. Representative images are given in Fig. 2A.

(*C*) Protein levels of HIF1α N803OH, HIF1α and FIH in U2OS cells treated with DMOG (1 mM), IOX1 (1 mM), IOX2 (0.25 mM), FG2216 (0.25 mM), VGB10B/IOX4 (0.05 mM) or DFO (0.5 mM) for 3 hours. β-tubulin was used as a loading control.

(*D*) Immunofluorescence staining of FIH (green) in U2OS cells treated with DMOG (1 mM), IOX1 (1 mM), IOX2 (0.25 mM), FG2216 (0.25 mM), VGB10B/IOX4 (0.05 mM) or DFO (0.5 mM) for 3 hours. TO-PRO-3 (blue) was used to stain nuclei. Scale bar: 20 μm.

Journal of Cell Science • Supplementary information

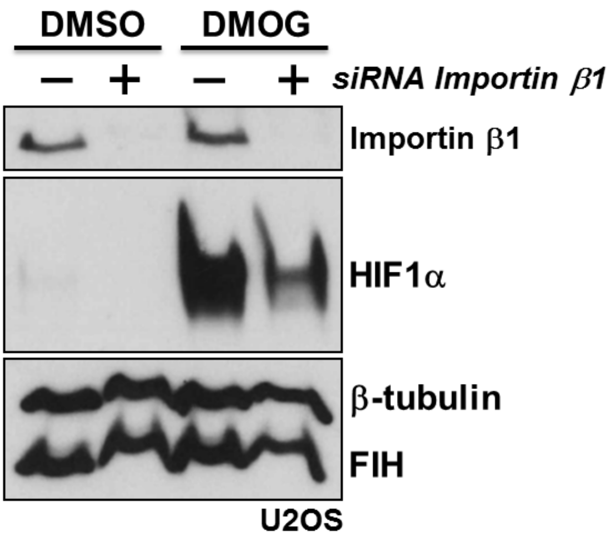

Supplementary Figure 3 - FIH complexes with importin β1 via HIF1α for nuclear import.

Protein levels of importin β1, HIF1α and FIH in U2OS cells with indicated treatments. U2OS cells were transfected with control siRNA or importin β1 siRNA for 3 days, followed by treatment with DMSO or DMOG (1 mM) for 3 hours. β-tubulin was used as a loading control.

Journal of Cell Science • Supplementary information

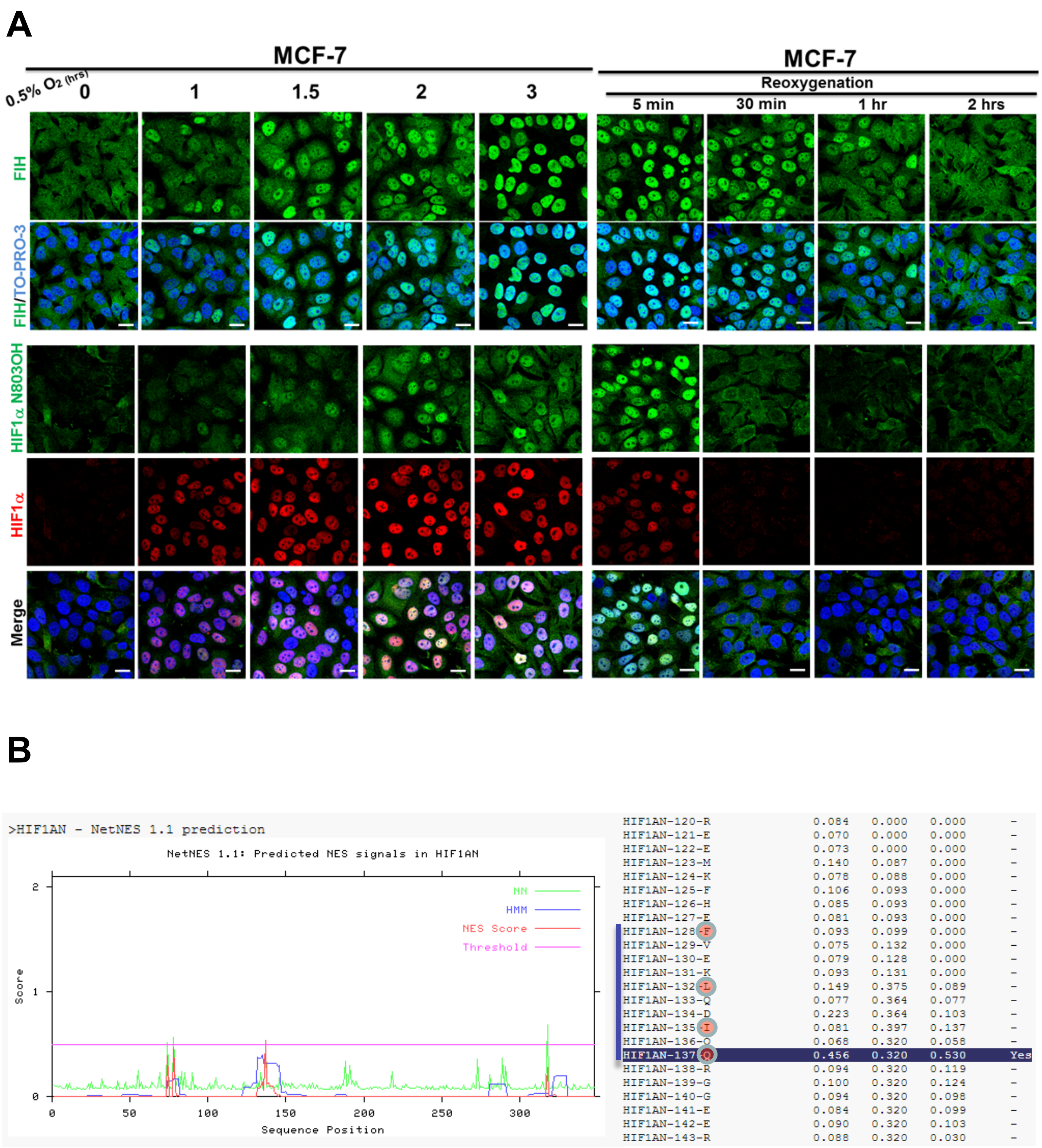

Supplementary Figure 4 - FIH exits the nucleus via a Leptomycin B-sensitive exportin 1 (CRM1)-dependent pathway.

(*A*) Immunofluorescence staining of FIH (green), HIF1α N803OH (green) and HIF1α (red) in MCF7 cells with the indicated hypoxia (0.5% O_2) and reoxygenation treatments. TO-PRO-3 (blue) was used to stain the nuclei. Scale bar: 20 µm.

(*B*) A nuclear export signal (NES) is predicted within FIH (amino acid 128-137) by NetNES (http://www.cbs.dtu.dk/services/NetNES/).

Journal of Cell Science • Supplementary information
